# Prevalence and clinical predictors of inappropriate direct oral anticoagulant dosage in octagenarians with atrial fibrillation

**DOI:** 10.1007/s00228-022-03286-2

**Published:** 2022-02-09

**Authors:** Andreina Carbone, Francesco Santelli, Roberta Bottino, Emilio Attena, Carmine Mazzone, Valentina Parisi, Antonello D’Andrea, Paolo Golino, Gerardo Nigro, Vincenzo Russo

**Affiliations:** 1 Chair of Cardiology, Department of Translational Medical Sciences, University of Campania “Luigi Vanvitelli”, Monaldi Hospital, P.Zzale Ettore Ruggeri, 80131 Naples, Italy; 2 Department of Political Sciences, University of Naples Federico II, Naples, Italy; 3 Department of Cardiology, Health Authority Naples 2 North, Naples, Italy; 4 Cardiovascular Centre, Health Authority, Trieste, Italy; 5 Department of Translational Medical Sciences, University of Naples Federico II, Naples, Italy; 6 Unit of Cardiology and Intensive Coronary Care, Umberto I Hospital, Nocera Inferiore, Italy

**Keywords:** Direct oral anticoagulant, Inappropriate dosage, Elderly, Atrial fibrillation, Stroke prevention

## Abstract

**Purpose:**

Older age is associated with inappropriate dose prescription of direct oral anticoagulants. The aim of our study was to describe the prevalence and the clinical predictors of inappropriate DOACs dosage among octogenarians in real-world setting.

**Methods:**

Data for this study were sourced from the multicenter prospectively maintained Atrial Fibrillation (AF) Research Database (NCT03760874). Of the AF patients aged ≥ 80 who received DOACs treatment, 253 patients were selected. Participants were categorized as appropriate dosage, overdosage, or underdosage. Underdosage and overdosage were, respectively, defined as administration of a lower or higher DOAC dose than recommended in the EHRA consensus.

**Results:**

A total of 178 patients (71%) received appropriate DOACs dose and 75 patients (29%) inappropriate DOACs dose; among them, 19 patients (25.6%) were overdosed and 56 (74.4%) were underdosed. Subgroup analysis demonstrated that underdosage was independently associated with male gender [OR = 3.15 (95% IC; 1.45–6.83); p < 0.001], coronary artery disease [OR = 3.60 (95% IC 1.45–9.10); p < 0.001] and body mass index [OR = 1.27 (1.14–1.41); p < 0.001]. Overdosage was independently associated with diabetes mellitus [OR = 18 (3.36–96); p < 0.001], with age [OR = 0.76 (95% IC; 0.61–0.96; p = 0.045], BMI [OR = 0.77 (95% IC; 0.62–0.97; p = 0.043] and with previous bleedings [OR = 6.40 (0.7; 1.43–28); p = 0.039]. There wasn’t significant difference in thromboembolic, major bleeding events and mortality among different subgroups. Underdosage group showed a significatively lower survival compared with appropriate dose group (p < 0.001).

**Conclusion:**

In our analysis, nearly one-third of octogenarians with AF received an inappropriate dose of DOAC. Several clinical factors were associated with DOACs’ overdosage (diabetes mellitus type II, previous bleeding) or underdosage (male gender, coronary artery disease, and higher body mass index). Octogenarians with inappropriate DOACs underdosage showed less survival.

**Supplementary information:**

The online version contains supplementary material available at 10.1007/s00228-022-03286-2.

## Introduction

Direct oral anticoagulants (DOACs) are recommended in preference to VKAs for the stroke prevention in atrial fibrillation (AF) patients eligible for oral anticoagulation therapy [1], based on their favorable risk–benefit profile regardless of the patients’ age [2, 3]. In particular, DOACs showed higher net clinical benefit versus VKAs in octogenarians with AF both in trial [4–7] and in real-world setting [8, 9]. Inappropriate dosage is a relevant issue affecting up to 15% of AF patients receiving DOACs [10] and the older age was associated with DOACs dosage not in compliance with the current recommendation [1, 10–12].

Data about the clinical factors associated to inappropriate dose prescription of DOACs, both under and overdosage, among elderly patients are lacking. The aim of our study was to describe the prevalence and the clinical predictors of inappropriate DOACs dosage among octogenarians in real-world setting.

## Materials and methods

### Study population

This is a retrospective analysis of a prospectively collected database. Data were sourced from the prospectively maintained Atrial Fibrillation Research Database (NCT03760874), shared by three Italian Cardiologic Centers (Monaldi Hospital, Naples; University of Campania “Luigi Vanvitelli”, Naples; Maggiore Hospital, Trieste), which includes all AF patients followed by these centers. Trained personnel abstracted clinical, demographic, laboratory, and treatment data of these participants from the electronic health records. We identified 1053 AF patients aged ≥ 80 years and only patients that received a treatment with DOACs (n = 263) were selected. We excluded from analysis patients with less than 1 year of follow-up (n = 10).

Follow-up data were obtained through outpatient visits every 3 to 6 months. During the follow-up visits, the clinical status, occurrence of stroke, transient ischemic attack (TIA), systemic embolism (SE), major bleeding (MB) events or other side effects were assessed. Ischemic stroke, TIA, SE, MB, and minor bleeding were defined as reported previously [9]. Chronic kidney disease was defined as kidney damage or creatinine clearance (CrCl) < 60 mL/min/1.73 m^2^ for 3 months or more, irrespective of cause [13]. CrCl was estimated with Cockcroft-Gault formula [13]. Permanent, persistent and paroxysmal AF, hypertension and diabetes were defined according to international guidelines [1, 14, 15]. Ischemic coronary disease is referred to both chronic coronary syndrome and previous acute coronary disease [16]. Participants were categorized as appropriate dose, overdosed, or underdosed. Underdosage and overdosage were, respectively, defined as administration of a lower or higher DOAC dose than recommended in the European Heart Rhythm Association (EHRA) consensus [17].

### Endpoints

The primary endpoint was to evaluate the prevalence of inappropriate DOACs dosage and to describe the clinical factors associated with dosing errors among the study population.

The secondary endpoint was to determine the occurrence of thromboembolic events (a composite of stroke, TIA and SE), MB and case fatality between DOACs appropriate and inappropriate dosage groups.

### Statistical analysis

Descriptive statistics were performed: frequency and percentage were reported for the categorical variables, mean, standard deviation, median and interquartile range (IQR) were used to summarize continuous variables. Continuous variables were compared using t-tests or Mann–Whitney test, and categorical variables were compared using χ2 tests. Univariate and multivariate logistic regression was used to investigate factors independently associated with inappropriately dosage. The variables included in the multivariate model were male gender, body mass index (BMI), CrCl, paroxysmal AF, permanent AF, diabetes mellitus, acetyl salicylic acid and amiodarone therapy. We included variables with p < 0.05 by the univariable test as a candidate for the multivariable analysis, with a forward variable selection, testing the addition of each variable, and repeating this process until none improves the model to a statistically significant extent.

Multinomial logistic regression was performed to compare underdose and overdose groups to appropriate dose of DOACs group. A logit model, adjusted for age, chronic kidney disease and acetyl salicylic acid use, was used to analyze the outcomes. A Kaplan–Meier analysis and log-rank test were used to compare the event rates of endpoints over time for the three groups (appropriate, low and high inappropriate dose). A two-sided P value less than 0.05 was considered significant for all tests. All statistical analyses were performed using R studio (RStudio Team (2016). RStudio: Integrated Development for R. RStudio, Inc., Boston, MA, USA URL http://www.rstudio.com/.

### Compliance with Ethical Standards

- The authors declare that they have no potential conflict of interest. All authors certify that they have no affiliations with or involvement in any organization or entity with any financial interest or non-financial interest in the subject matter or materials discussed in this manuscript.
- Ethical approval was waived by the local Ethics Committee of Monaldi Hospital in view of the retrospective nature of the study and all the procedures being performed were part of the routine care.
  All procedures performed in studies involving human participants were in accordance with the ethical standards of the institutional and/or national research committee and with the 1964 Helsinki declaration and its later amendments or comparable ethical standards.
- Informed consent was obtained from all individual participants included in the study. Additional informed consent was obtained from all individual participants for whom identifying information is included in this article.

## Results

A total of 253 patients (median age 83 [4.70] years; 58% women) were included in the study. Demographic and clinical characteristics of the population are showed in Table 1. 178 patients (71%) received appropriate DOACs dose and 75 patients (29%) inappropriate DOACs dose; among them 19 patients (25.60%) were overdosed and 56 (74.40%) were underdosed. The CHA_2_DS_2_-VASc score of the overall population was 4.50 (± 1.20), whereas the HASBLED score was 2.90 ± 0.90. Permanent AF occurred in a lower percentage of patients with inappropriate dosing compared to appropriate dosing (44% vs 65%; p < 0.001); as did acetyl salicylic acid combination (4% vs 15.70%; p = 0.009), compared with appropriate dose group (Table 1).

**Table 1:** Baseline characteristics and clinical outcomes of study population

	**Appropriate dose (n = 178)**	**Inappropriate dose (n = 75)**	**p-value**
Age, median (IQR)	84.14 (4.70)	83.66 (4)	0.35
Women, n (%)	109 (61.20)	36 (48)	0.05
BMI (kg/m^2^) media (± SD)	24 (± 3.78)	26.50 (± 3.80)	0.50
CrCl (ml/min/m^2^), median (IQR)	45.50 (17)	54 (16)	< 0.001
Overdosage, n (%) Underdosage, n (%)		19 (25.30) 56 (74.70)	- -
Type of DOACs, n (%)			
Dabigatran Apixaban Rivaroxaban Edoxaban	66 (37) 37 (21) 73 (41) 2 (1)	0 (0) 27 (36) 48 (64) 0 (0)	- 0.01 < 0.001 -
Paroxysmal atrial fibrillation, n (%)	24 (13.50)	21 (28)	0.006
Permanent atrial fibrillation, n (%)	117 (65.70)	33 (44)	< 0.001
Persistent atrial fibrillation, n (%)	27 (15.20)	18 (24)	0.06
Coronary artery disease, n (%)	34 (19.10)	22 (29.30)	0.07
Diabetes mellitus II type, n (%)	32 (18)	22 (29.30)	0.06
Arterial Hypertension, n (%)	153 (86)	69 (92)	0.18
Chronic kidney disease, n (%)	71 (39.90)	34 (45.30)	0.42
Previous ischemic stroke, n (%)	40 (22.50)	18 (24)	0.79
Previous bleedings, n (%)	34 (19.10)	16 (21.30)	0.68
ASA, n (%)	28 (15.70)	3 (4)	0.009
Amiodarone, n (%)	11 (6.20)	11 (14.70)	0.03
**Outcomes**			
Stroke/TIA/SE, n (%)	7 (3.90)	2 (2.60)	0.62
Minor bleedings, n (%)	5 (2.80)	3 (4)	0.6
Major bleedings, n (%)	7 (3.93)	5 (6.60)	0.35
Death, n (%)	31 (17)	15 (20)	0.60

*IQR* interquartile range, *BMI* body mass index, *CrCl* creatinine clearance, *DOACs* direct oral anticoagulants, *SD* standard deviation, *ASA* acetyl salicylic acid, *SE* systemic embolism, *TIA* transient ischemic attack

**Fig. 1:**
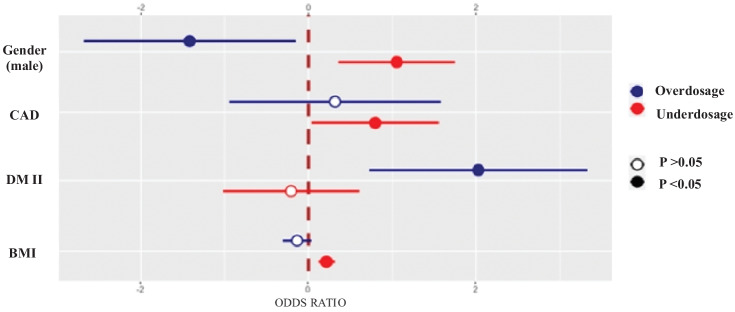
Forest plot shows the association of male gender, CAD, DM II, and BMI with the risk of DOAC overdosage and/or underdosage

At multivariate logistic regression analysis, the lower rate of permanent AF [OR = 0.45 (95% IC; 0.22–0.91); p = 0.03], and the lower combination therapy with acetyl salicylic acid [OR = 0.19 (0.05–0.71); p = 0.01] were independently associated with inappropriate dose prescription (Table 2). Subgroup analysis dividing the inappropriate DOACs dose group in underdosage and overdosage subgroups demonstrated that underdosage was independently associated with male gender [OR = 3.15 (95% IC 1.45–6.83); p < 0.001], coronary artery disease [OR = 3.60 (95% IC 1.41–9.10); p < 0.001] and BMI [OR = 1.27 (1.14–1.41); p < 0.001] (Table 3; Fig. 1). Overdosage was independently associated with age [OR = 0.76 (95% IC; 0.61–0.96; p = 0.045], male gender [0.19 (0.05–0.84); p = 0.021], diabetes mellitus [OR = 18 (3.36–96); p < 0.001], BMI [OR = 0.77 (0.11; 0.62–0.97; p < 0.043] and with previous bleedings [OR = 6.40 (0.70; 1.43–28), p = 0.039].

**Table 2:** Association between patients’ characteristics and inappropriate dose prescription of DOACs

	**Univariate** **OR (95% CI), p value**	**Multivariate** **OR (95% CI), p value**
Male gender	1.70 (0.90–2.90); p = 0.05	1.19 (0.5–2.8); p = 0.60
BMI	1.13 (1.05–1.20); p = 0.001	1.06 (0.9–1.1); p = 0.20
CrCl	1.04 (1.02–1.07); p < 0.001	1.03 (0.90–1.06); p = 0.30
Paroxysmal atrial fibrillation	2.40 (1.20–4.80); p = 0.007	1.27 (0.50–1.90); p = 0.57
Permanent atrial fibrillation	0.41 (0.21–0.72); p = 0.002	0.45 (0.22–0.91); p = 0.03
Diabetes mellitus II type	1.89 (1.01–3.50); p = 0.04	1.39 (0.68–2.80); p = 0.36
ASA	0.22 (0.65–0.75); p = 0.016	0.19 (0.05–0.71); p = 0.014
Amiodarone	2.60 (1.07–6.20); p = 0.035	1.50 (0.50–4.20); p = 0.40

*BMI* body mass index, *CrCl* creatinine clearance, *SE* systemic embolism, *TIA* transient ischemic attack, *ASA* acetyl salicylic acid, *OR* odds ratio, *IC* interval confidence

**Table 3:** Clinical predictors of underdosage and overdosage DOAC prescription among study population

**Variable**	**Overdose** **OR (SE, CI 95%); p value**	**Underdose** **OR (SE, CI 95%); p value**
Age	0.76 (0.11; 0.61–0.96) p = 0.04	1.03 (0.06; 0.91–1.17) p = 0.15
Gender (male)	0.19 (0.74; 0.05–0.84) P = 0.02	3.15 (0.39; 1.45–6.83) P < 0.001
BMI	0.77 (0.11; 0.62–0.97) P = 0.04	1.27 (0.05; 1.14–1.41) P < 0.001
Coronary artery disease	4.30 (0.80; 0.88–20.70) p = 0.24	3.60 (0.47; 1.41–9.10) P < 0.001
Diabetes mellitus type II	18 (0.80; 3.36–96) P < 0.001	0.78 (0.40; 0.33–1.86) p = 0.11
Arterial hypertension	5.10 (1.30; 0.40–64) p = 0.23	1.02 (0.60; 0.30–3.53) p = 0.19
Chronic kidney disease	1.20 (0.60; 0.38–3.87) p = 0.37	1.15 (0.40; 0.53–2.51) p = 0.39
Stroke/TIA/SE	0.36 (0.77; 0.08–1.78) p = 0.15	1.06 (0.44; 0.44–2.55) p = 0.27
Previous bleedings	6.40 (0.70; 1.43–28) P = 0.03	0.90 (0.46; 0.40–2.45) p = 0.11
Amiodarone	0.76 (1.30; 0.06–9.72) p = 0.11	2.70 (0.60; 0.87–8.74) p = 0.17

*BMI* body mass index, *SE* systemic embolism, *TIA* transient ischemic attack, *OR* odds ratio, *IC* interval confidence

### Clinical outcomes

**Fig. 2:**
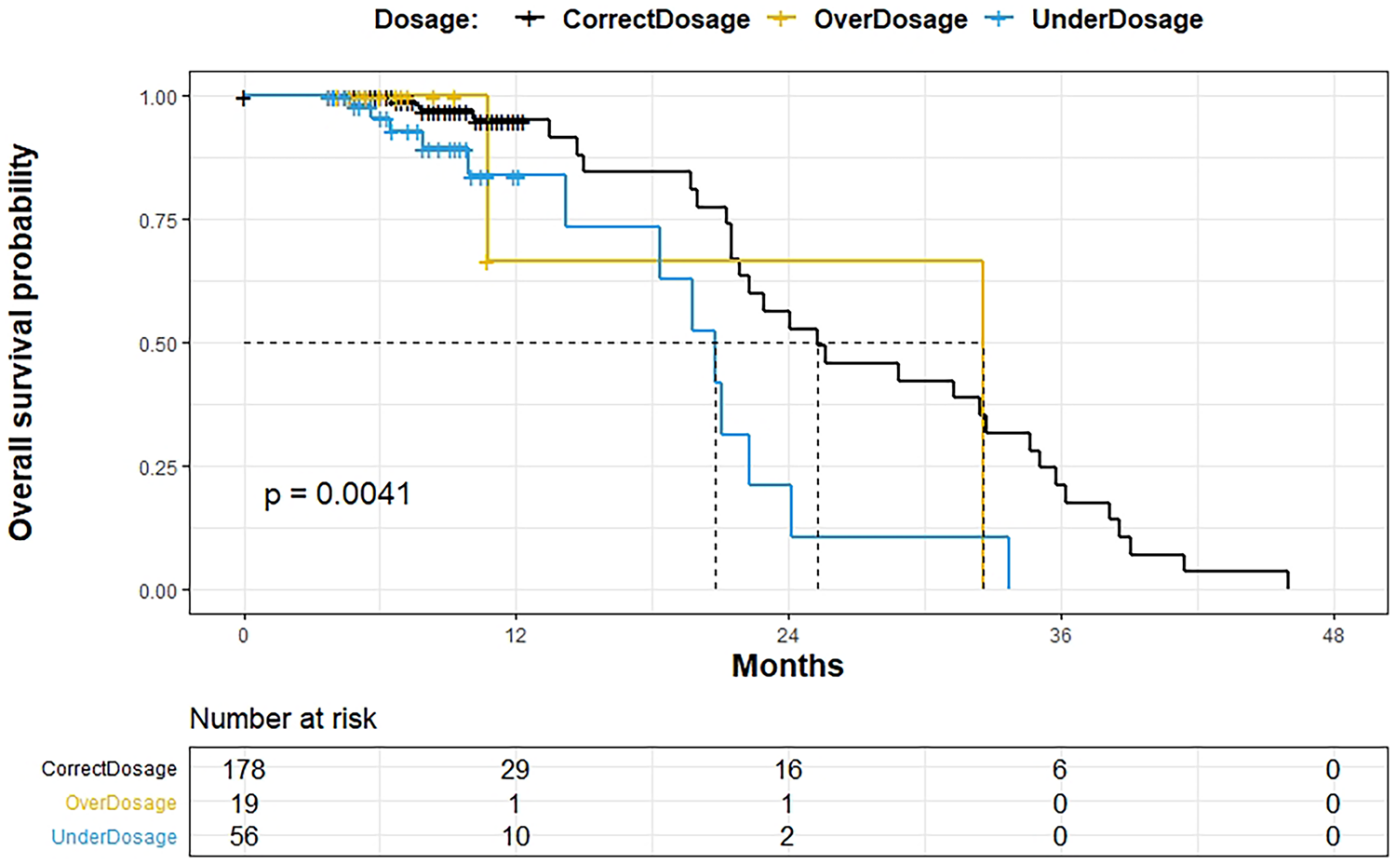
Kaplan–Meier curves comparing survival rate in appropriate dosage, overdosage, and underdosage DOACs subgroups

Over a mean follow-up of 32 ± 10 months, a total of 46 patients (31 in appropriate vs 15 in inappropriate group; 17% vs 20%, respectively; p = 0.6) died; no statistically significant difference was found among the subgroups (appropriate, underdosage and overdosage) (Sup). Figure 2 shows the Kaplan–Meier cumulative probability of survival, respectively, in appropriate, underdosage and overdosage groups (p = 0.004). Underdosage group showed a significatively lower survival compared with appropriate dose group (p < 0.001).

12 patients (7 in appropriate dose vs 5 in inappropriate group, p = 0.3) had MB events; 9 patients (7 in appropriate dose vs 2 in inappropriate group, p = 0.6) experienced thromboembolic events during the follow-up. At subgroup analysis (appropriate, underdosage and overdosage) there wasn’t significant difference in both thromboembolic and MB events among the groups (Supplementary Table S1).

Multivariate regression analysis, related to patients’ characteristics, clinical outcomes and mortality among study population, is shown in (Supplementary Table S2). According to this model, male gender [OR = 2.10 (95% IC; 1.08–4.20); p = 0.02], BMI < 18.50 kg/m^2^ [OR = 5 (95% IC; 1.20–21); p = 0.02], and SE/TIA [OR = 4 (95% IC 1.01–17); p = 0.048] were independently associated with death.

## Discussion

The main finding of our study can be summarized as follows: 29% of octogenarians with AF were treated with inappropriate dose of DOACs in real world setting; in particular, the prevalence of underdosage and overdosage was 22% and 7.50%, respectively.

The lower rate of permanent AF and the lower combination therapy with acetylsalicylic acid were the only independent predictors of inappropriate DOACs’ dosage; these findings could be related to greater attention in prescribing the correct dosage in elderly patients with permanent AF and in case of association with antiplatelet agents. The DOAC’s inappropriate dosage did not seem correlated to the incidence both thromboembolic and MB events; however, the underdosage group showed a significatively lower survival compared with appropriate dose group.

Previous studies including younger AF cohorts on DOACs therapy reported a prevalence of inappropriate underdosage ranging from 14 to 45% [12, 18–22] and a prevalence of overdosage ranging from 2.40 to 14% [10–12]; our data confirmed these prevalences among octogenarians with AF.

According to the analysis of Sugrue et al. [10], the underdosage was independently associated with male gender. Other studies, including younger patients, showed female subjects received more frequently inappropriate DOACs dose [12]. Furthermore, we found an association between DOAC inappropriate underdosage and coronary artery disease. In the Global Anticoagulant Registry in the FIELD-AF (GARFIELD-AF) [23], which included 10,426 patients receiving DOACs, underdosage were associated to acute coronary syndrome, female sex, non-Caucasian ethnicity, vascular disease, prior stroke and diabetes.

Similarly, to the analysis by Ruiz et al. [12], our analysis identified higher BMI as an independent predictor of inappropriately low dose prescription of DOACs among octogenarians with AF. Patients with increased BMI should receive standard doses of DOAC, so the main risk of wrong prescription in elderly AF patients with obesity is to receive an inappropriately low dose and not a high dose. BMI evaluation has some limitations in very elderly patients, due to sarcopenia and the possible different references values in this population [24]. Furthermore, low body weight (≤ 60 kg) is one of the criteria for reduced DOAC dose [17], but it’s possible that many physicians don’t take it into account when adjusting DOAC dose in the elderly with increased BMI.

In our analysis age, gender male and BMI were negatively associated with overdosage; in particular, younger patients, female and those with lower BMI would be more likely to have an inappropriate overdosage prescription among octogenarians with AF. Moreover, we showed that diabetes mellitus was an independent predictor of inappropriate DOACs’ overdosage, according to previous evidence [10]. This data might be explained by the increased risk of ischemic cerebrovascular disease or stroke among AF patients with diabetes [25]. The combination of diabetes and AF is associated with increased risk for death and major cerebrovascular deaths [26]. Diabetes and AF are independently associated with platelet and fibrinogen activation contributing to changes in the blood constituents and to thrombus formation [27]. Furthermore, previous bleeding was positively associated with inappropriate DOACs’ overdosage.

### Inappropriate DOAC dosage and clinical outcomes

The inappropriate dose prescription of DOACs has important clinical implications, in terms of thromboembolic events and bleedings. Yao et al. [28] reported higher risk of stroke among patients who received an inappropriate underdosage of apixaban; Steinberg et al. [29] showed that DOACs overdosage was significantly associated with increased risk of all-cause mortality, and DOACs underdosage was associated with increased risk of cardiovascular hospitalization.

Sugrue et al. [10] reported an increased incidence of stroke, SE and bleedings among patients with inappropriate DOACs dosage; however, it was not statistically significant, probably for the lower event rate and short duration of follow-up that may have limited the power to detect any significant differences in outcomes.

In the GARFIELD-AF [23], the non-recommended dose (underdosage and overdosage combined) was associated with an increased risk of all-cause mortality; conversely, both the ischemic stroke and major bleeding risk was not significantly different irrespective of dosing levels, although underdosed patients had a significantly lower risk of bleeding.

We did not find any significant association between inappropriate dose prescription of DOACs, both overdosage and underdosage, and the incidence of thromboembolic events, major bleedings or case fatality among octogenarians with AF; our result could be explained by the age of our population and the short follow-up.

Among our study population, the male gender, BMI < 18.50 kg/m^2^, and thromboembolic events (stroke/TIA and systemic embolisms) were the only independent predictors of mortality.

Previously, Deng et al. [30] showed a higher risk of in-hospital mortality in underweight AF population, compared with those who were overweight and in patients with a thromboembolic event. Furthermore, many studies on AF and cardiovascular mortality have reported favorable outcomes for patients with higher BMI, the so called “obesity paradox” [8, 31–34].

We didn’t show significant difference in case fatality among the three groups (overdosage, underdosage and appropriate dose); however, according to the survival analysis, the underdosage group showed lower survival compared to the others, with more early deaths.

### Limitations

The small size of our study population, the short-term follow-up, and the relatively low incidence of clinical endpoints did not allow a subgroup analysis according to DOACs type. The low incidence of some clinical events did not allow to perform a multivariate analysis.

No specific information on adherence and persistence to the therapy was available, although patients’ compliance was assessed during follow-up visits and those few patients who had definitely stopped DOACs were excluded.

Despite these limitations, this is the first analysis of inappropriate DOACs dose prescription among very elderly patients (≥ 80 years). Further larger studies are needed to better underline the clinical drivers and the outcomes associated to inappropriate underdosage and overdosage in this setting.

## Conclusion

In our multicenter registry analysis, nearly one-third of octogenarians with AF treated with DOACs received an inappropriate dose. Octogenarians with diabetes mellitus and previous bleeding could have an increased risk of overdosage, whereas age, male gender, and higher BMI are negatively associated with it. Furthermore, male patients with coronary artery disease and higher BMI could have an increased risk to receive an underdosage prescription. In our analysis, the underdosage group showed a significantly lower survival compared with that of the appropriate dose group. Further research on the effectiveness of clinical interventions to address inappropriate dosing in very elderly patients would be valuable.

## Supplementary information

Below is the link to the electronic supplementary material.

Supplementary file1 Difference in all-cause of mortality, stroke/systemic embolism/TIA and major bleedings between appropriate, underdosage and overdosage DOAC prescription. (DOC 32 KB)

Supplementary file2 Association between patients’ characteristics, clinical outcomes and case fatality among study population. (DOC 38 KB)

## Data Availability

Data available on request from the authors. The data that support the findings of this study are available from the corresponding author, [V. R.], upon reasonable request.
