# Supplementary material for: Prevalence and clinical predictors of inappropriate direct oral anticoagulant dosage in octagenarians with atrial fibrillation
Source: Eur J Clin Pharmacol. 2022 Feb 9;78(5):879–86. doi: 10.1007/s00228-022-03286-2 (PMC9005392; doi:10.1007/s00228-022-03286-2)
Supplement: Supplementary file 1 — Supplementary file1 Difference in all-cause of mortality, stroke/systemic embolism/TIA and major bleedings between appropriate, underdosage and overdosage DOAC prescription. (DOC 32 KB) [file 228_2022_3286_MOESM1_ESM.doc]

|  | **Total** | **All-cause of mortality** | | | **Stroke/systemic embolism** | | | **Major bleedings** | | |
| --- | --- | --- | --- | --- | --- | --- | --- | --- | --- | --- |
|  | n | n (%) | OR [95% CI] | p-value | n (%) | OR [95% CI] | p-value | n (%) | OR [95% CI] | p-value |
| Appropriate dosage | 178 | 67.40 | - | - | 77.80 | - | - | 58.30 | - | - |
| Underdosage | 56 | 28.30 | 1.44 [0.67-2.96] | 0.34 | 11.10 | 0.50 [0.02-2.97] | 0.49 | 25 | 1.42 [0.28;5.45] | 0.60 |
| Overdosage | 19 | 4.35 | 0.59 [0.08;2.25] | 0.48 | 11.10 | 1.51 [0.06;9.47] | 0.73 | 16.70 | 2.99 [0.38;14] | 0.25 |

Abbreviations: OR=odds ratio; CI= confidence interval.

**Supplement table 1. Difference in all-cause of mortality, stroke/systemic embolism/TIA and major bleedings between appropriate, underdosage and overdosage DOAC prescription.**
