# Supplementary material for: Prevalence and clinical predictors of inappropriate direct oral anticoagulant dosage in octagenarians with atrial fibrillation
Source: Eur J Clin Pharmacol. 2022 Feb 9;78(5):879–86. doi: 10.1007/s00228-022-03286-2 (PMC9005392; doi:10.1007/s00228-022-03286-2)
Supplement: Supplementary file 2 — Supplementary file2 Association between patients’ characteristics, clinical outcomes and case fatality among study population. (DOC 38 KB) [file 228_2022_3286_MOESM2_ESM.doc]

|  | **Univariate**  **OR (95% IC; p-value)** | **Multivariate**  **OR (95% IC; p-value)** |
| --- | --- | --- |
| Underdosage | 1.50 (0.70-3); p=0.20 | - |
| Overdosage | 0.50 (0.10-2); p=0.50 | - |
| Male gender | 1.70 (0.90-3.30); p=0.07 | 2.10 (1.10-4.20); p=0.02 |
| Weight ≤ 60 Kg | 1.70 (0.80-3.50); p= 0.12 | - |
| BMI | 0.90 (0.80-0.99); p=0.03 | - |
| BMI<18,5 kg/m^2^ | 3.80 (0.90-14); p=0.05 | 5 (1.20-21); p=0.02 |
| CrCl | 0.98 (0.96-1); p=0.30 | - |
| CrCl <45 ml/min | 1.30 (0.70-2.60); p= 0.30 | - |
| Stroke/SE/TIA | 3.80 (0.90-14); p=0.05 | 4 (1.01-17); p=0.04 |
| Major bleedings | 2.30 (0.60-8); p= 0.17 | 3.06 (0.80-11); p=0.08 |

**Supplements table 2. Association between patients’ characteristics, clinical outcomes and case fatality among study population.**

BMI= body mass index; CrCl= creatinine clearance; SE=systemic embolism; TIA= transient ischemic attack; OR= odds ratio; IC= interval confidence.
